# Comparison between ozone and CHX gel application for reduction of pain and incidence of dry socket after lower third molar removal

**DOI:** 10.1002/cre2.675

**Published:** 2022-10-17

**Authors:** Jehona Ahmedi, Zana Agani, Resmije Ademi Abdyli, Mergime Prekazi Loxha, Vjosa Hamiti‐Krasniqi, Aida Rexhepi, David Stubljar

**Affiliations:** ^1^ Department of Oral Surgery University of Prishtina Prishtina Kosovo; ^2^ Department of Maxillofacial Surgery University of Prishtina Prishtina Kosovo; ^3^ UBT College Prishtina Kosovo; ^4^ In‐medico, Department of Research and Development Metlika Slovenia

**Keywords:** chlorhexidine, dry socket, efficacy, ozone gas, third molars

## Abstract

**Objectives:**

The aim of this study is to evaluate the efficacy between ozone gas and 1% chlorhexidine (CHX) gel in the incidence of dry socket after surgical extraction of impacted lower third molars.

**Material and Methods:**

Overall, 30 patients of both genders were included in the study, with indication of surgical extraction of lower third molar, positioned similarly after being clinically and radiographically checked by X‐ray and orthopantomography. Each patient was subjected to both groups in separate sessions: treated with ozone gas and with CHX gel 1%. Data on pain intensity, number of taken analgesics‐painkillers, and dry socket were recorded for 48 h and at Day 7.

**Results:**

Ozone gas and CHX gel effectively reduced pain intensity and prevented dry socket. The number of taken analgesics 48 h and 7 days after surgery showed no statistical significance. The same was observed for the distribution of pain. Only one patient reported the occurrence of dry socket 7 days after the surgical extraction.

**Conclusions:**

Ozone gas and CHX 1% gel are both efficient in decreasing postoperative symptoms and incidence rates of dry socket, but in comparison to each other, the use of ozone gas is showing a bit better prevention capability.

## 1 INTRODUCTION

Surgical extraction of third molars from lower jaw represent very challenging surgical procedure, which often requires additional interventions. Common postoperative complications are for instance dry socket and followed by high‐intensity pain as the main symptoms that are directly associated with the procedure of removing mandibular third molars. Studies have addressed this problem, but in the absence of etiology the prevention has been considered as doubtable (Filipovic‐Zore et al., 2011; Ribeiro et al., 2007). Etiologically it was shown that bacterial infection and trauma of tooth socket during and after extraction are factors that trigger the appearance of dry socket (Catellani, 1979; Nitzan et al., 1978; Peñarrocha et al., 2001; Rozanis et al., 1977).

Dry sockets normally occur when a clot detaches from the extraction site before the wound has healed. It causes severe pain 1–3 days after extraction, which may delay the healing and can even progress to chronic bone infection. The incidence of dry socket is 3% for all teeth and more than 30% in case of lower third molars (Otake et al., 2021). Disintegrated clot and pain are considered the most important clinical and subjective symptoms of dry socket. The frequency and intensity of pain varies individually depending on the threshold of pain tolerance in patients. Based on the clinical picture. Hermesch et al. (1998) classified dry socket into three types: alveolitis superficialis marginalis with inflamed and painful mucosa around the alveolus of the tooth; alveolitis suppurativa in cases where it comes to the infection of the coagulum which is covered by a greenish‐gray membrane accompanied by local pain and temperature; and dry socket with partial or total loss of the coagulum leading to the exposure of the alveolar walls, fetor ex‐ore, and intense pain that is resistant even to analgesics. Oikarinen (1989) classified this complication in two forms; true alveolitis with more sensational symptoms which requires professional clinical treatment and nonspecific alveolitis which is accompanied by pain of low intensity which is managed with analgesics and does not require additional intervention by the dentist. Despite different and controversial opinions regarding the naming, classification, and clinical description of this complication, in general, is characterized as inflammation of the socket, where the intensity of the pain and the time of its onset remain specific clinical signs indicative for diagnosis.

Alleviating symptoms such as pain was a focus area of many studies, which studied wound closure techniques, the effect of analgesics (Adeyemo et al., 2006), corticosteroids (Makkar & Makkar, 2011), and antibiotics (Jaffar & Tin, 2009; Khan et al., 2010; O'brien et al., 2005). In the literature we can found good results for the use of chlorhexidine (CHX) before or after surgical extraction of third molars. The use of CHX significantly reduces postoperative complications and symptoms including the reduction of dry socket (Costa et al., 2013; Daly et al., 2012; Filipovic‐Zore et al., 2011; Lambert et al., 1997; Loncar et al., 2009). Moreover, CHX in clinical practice also reduced the use of other antiseptics (Caso et al., 2005; Fedorowicz et al., 2008; Hermesch et al., 1998).

The methods of prevention included administering CHX into the extraction socket or rinsing the mouth with CHX (Eick et al., 2011). However, the disadvantage is that CHX can easily flow out of the socket or rinsing of the mouth with CHX can lead to the loss of blood clots. Therefore, studies have focused to find the treatments which involved inserting ointment containing antibacterial or steroidal agents into the extraction socket. For instance, chlortetracycline ointment after tooth extraction showed a preventive effect on dry socket, but not on pain (Otake et al., 2021).

Photobiomodulation therapy was tested for the prevention of dry socket occurrence and postoperative pain that are following third molar surgery. It reduced the incidence of dry socket. The incidence of dry socket typically occurs 3–7 days post‐extraction, and results suggest that treatment within the 7 days could help reduce the risk of dry socket development (Nejat et al., 2021).

Ozone has documented antimicrobial capabilities (Dyas et al., 1983). Beside CHX the use of ozonated water accelerated would healing of epithelia already established in first 2 days after the surgery, even without any need for systemic therapy (Dyas et al., 1983). Usage of ozone reduced the inflammation and pain, and stimulated healing through synthesis of interleukins, leukotrienes, and prostaglandins which are acting anti‐inflammatory (Adeyemo et al., 2006). Alongside alleviating the inflammation, ozone also stimulates the secretion of nitroglycerin, which acts as vasodilator of arterioles and activates angiogenesis in the inflamed tissue (Ebensberger et al., 2002; Khan et al., 2010).

Therefore, the aim of the current study was to evaluate and compare the efficacy between ozone gas and conventional CHX gel for occurrence of dry socket after surgical extraction of impacted mandibular third molars in order to justify their preventive use in treatment protocols.

## 2 MATERIAL AND METHODS

This prospective study was designed as split‐mouth research and was conducted at the Oral Surgery Clinic at the University Dental Clinical Center of Kosovo. The research was conducted with the permission of the Ethics Committee from Faculty of Medicine (Decision No. 1551, dated 30.03.2010) and performed with full compliance and the principles of medical ethics and good practice while respecting the Helsinki Declaration on Human Research. Prior to the surgical intervention, the patients were provided with the consent form for cooperation through which they were informed about the surgical intervention procedure and the purpose of the research. Initially, the criteria for inclusion, noninclusion, and eventual exclusion of patients from the study were selected, which criteria were strictly observed during the selection of patients.

### 2.1 Data collection

The selection of patients was made within outpatient clinics, which referred patients to the Oral Surgery Clinic for surgical extraction of the third molars of the lower jaw. This clinical study included patients of both sexes, age over 18 years, who were presented for surgical extraction of third impacted molars of the lower jaw, in a symmetrical position in two separate sessions (14 days in between). After anamnesis, clinical and radiographic examination (orthopantomography) the indications for their extraction of both molars were evaluated and determined.

Criteria for including patients into research:

(a) Patients with third impacted bilateral molars, symmetrically in the same position, position evaluated by radiography.
(b) Patients in whom operative extraction of third molars is indicated with the creation of a mucoperiosteal flap.
(c) Patients regardless of gender.

Criteria for noninclusion of patients into research:

To avoid the possibility of influencing the surgical procedure as well as the postoperative situation were not included:

(a) Patients who have a contraindication for surgery.
(b) Patients with chronic systemic diseases.
(c) Patients receiving immunosuppressive therapy.
(d) Female patients with pregnancy and lactation,
(e) Female patients using oral contraceptives.
(f) Female patients with menstrual cycle on the day of intervention.
(g) Patients who smoked.
(h) Patients with a history of allergy to chlorhexidine, lidocaine, paracetamol or ibuprofen.
(i) Patients requesting the extraction of two‐third molars in the same session.

Criteria for excluding patients from the research:

(a) Voluntary exclusion of the patient.
(b) Lack of cooperation in any of the work phases.

Each patient received a card with general and anamnestic data. Dental status including clinical data before the surgery, tooth position, ratio to the second molar, ratio to ramus mandibulae, and indication for extraction were recorded. The second part of the card was containing data during the intervention such as severity of extraction difficulty and duration of intervention. The last part of the card summarized the data that were recorded 48 h and 7 days after the intervention such as pain, number of analgesics consumed, and dry socket 48 h and 7 days after extraction.

Clinical examination was performed by the oral surgeon to assess the fulfillment of the criteria for inclusion. After setting the indication for extraction of both lower jaw molars and after verifying the similar position of the teeth on both sides, each patient was categorized for the application of CHX 1% in gelatinous state on one side, and application of gaseous ozone for a period of 12 s with the Prozone apparatus on the other side of jaw. Third molars were extracted in two separate sessions, once applied with CHX and secondly with ozone gas. The advantage of this paper is that the same patient was the subject of both groups.

The patient was not aware to which group he/she belongs in order not to suggestively reflect on the therapy and impair the results due to subjectivism. Altogether, 30 patients were subjected for the study and a total of 60 third molars of the lower jaw were extracted.

### 2.2 Surgical procedure

Every surgical intervention was performed by the same oral surgeon, using the same surgical technique on both sides to minimize the discrepancies of the surgical approach in the tissues of the oral cavity, and thus to minimize the impact of trauma as a possible factor for the appearance of postoperative symptoms.

Surgery was done under local anesthesia Lidocaine 2% 1:80000 2 ml, and the same triangular flap was used to all interventions in all patients. After elevating the mucoperiosteal flap and bone exposure, the tooth was extracted. The same flap was applied in all interventions to all groups of patients (triangular flap). No. 15 surgical scalpel was used for the incision of the triangular flap. After elevation of the mucoperiosteal bulb with a 16 cm Williger Raspator, the exposed bone was eroded with a tungsten carbide no. 3 round bur with a surgical insert (W&H). Depending on the position and as needed, the crown or the roots of the tooth were also separated with tungsten carbide fissural bur no. 1702. Extraction of the third molar from the socket and flattening of the roughness on the marginal blades of the alveolus, tungsten carbide no. 8 round bur. The procedure of applying gaseous O3 for 12 s with the Prozone device, CHX 1% gel (Chlorhexamed 1%) 1 ml was performed depending on the group.

The procedure of applying gas ozone was performed for 12 s with the Prozone device, or 2 ml of 1% CHX digluconate gel (Chlorhexamed 1%) was applied. The wounds were placed by individual sutures with 4‐0 (Ethicon Vicryl, 4/0 absorbable sutures, 19 mm rev cut 3/8, 45 cm).

Postoperative care was cold pack from outside. No food was recommended for 2 h. After that cold and soft food was recommended but jewed in the opposite side of the surgical site. The first 24 h patients did not rinse their mouth and afterwards rinsing was recommended only with chamomile tea or salted water.

The first checkup was 48 h after the intervention and the second checkup on the seventh day after the intervention. Pain was assessed according to VAS (visual analog scale) based on NIPC (National Initiative on Pain Control) which provides assessment of pain intensity and quality by the patient. The number of analgesics—the number of tablets consumed (ibuprofen 400 mg) was recorded in the first 48 h after tooth extraction and until the seventh day. Incidence of dry socket was recorded as well.

### 2.3 Statistics

The data analysis was performed using a statistical program SPSS 21 (IBM, New York, USA). Variables of pain intensity and appearance of dry socket were presented as frequencies. The differences 48 h after the surgery extraction and 7 days after the surgery extraction were tested with Pearson's *χ* ^2^ test. Differences continuous variables were tested with the Wilcoxon matched pairs test (*Z*). Significance for all tests was determined by *p* < .05.

## 3 RESULTS

Overall, 30 patients were enrolled that had surgical extraction of third semi‐impacted and/or impacted molars of the lower jaw, in a symmetrical position. The differences in the presence of taken analgesics and pain 48 h after extraction of the third molars of the lower jaw are shown in Table 1.

**Table 1:** Differences among tested groups 48 h after surgical procedure

	Ozone gas (*n* = 30)	1% Chlorhexidine gel (*n* = 30)	*p* Value
Number of analgesics	84,85	98,15	0.330^a^
Pain intensity			0.370^b^
No pain (VAS 0)	9 (30.0%)	6 (20.0%)	
Lower pain intensity (VAS 1–3)	19 (63.3%)	17 (56.7%)	
Pain with moderate intensity (VAS 4–6)	2 (6.7%)	6 (20.0%)	
High intensity pain (VAS 7–10)	0	1 (3.3%)	

^a^ Wilcoxon matched pairs test (*Z*‐test).

^b^ Pearson's *χ* ^2^ test.

The number of taken analgesics 48 h after surgery shows more tablets in Group 2, but with no statistical significance when compared to Group 1. Moreover, in the displayed distribution of pain 48 h after extraction there is no significant difference between the two groups. In patients applied with ozone gas, out of a total of 30 patients none of them had declared high intensity pain and 9 (30.0%) did not report pain. While in patients with 1% CHX gel out of 30 patients, 1 (3.3%) had declared high intensity pain with VAS (7–10) and 6 (20.0%) moderate intensity (VAS 4–6).

Seven days after surgery more patient took analgesics from the group with 1% CHX gel than from the group with ozone gas, but the difference was not statistically significant (Table 2). The differences in pain intensity 7 days after extraction displayed no significant difference between the two groups. Only one patient reported the occurrence of dry socket 7 days after the surgical extraction of the third molars of the lower jaw, but this was also not statistically relevant when comparing the two groups (Table 2). In alveola where ozone gas was applied no case of alveolitis was presented while in the CHX group 1 patient was diagnosed with an occurrence of dry socket.

**Table 2:** Differences among tested groups 7 days after surgical procedure

	Ozone gas (*n* = 30)	1% Chlorhexidine gel (*n* = 30)	*p* Value
Number of analgesics	91,10	99,10	0.950^a^
Pain intensity			0.470^b^
No pain (VAS 0)	19 (63.3%)	19 (63.3%)	
Lower pain intensity (VAS 1–3)	11 (36.7%)	9 (30.0%)	
Pain with moderate intensity (VAS 4–6)	0	2 (6.7%)	
High intensity pain (VAS 7–10)	0	0	
Dry socket	0	1 (3.3%)	1.000^b^

^a^ Wilcoxon matched pairs test (*Z*‐test).

^b^ Pearson's *χ* ^2^ test.

The case of dry socket was treated by curettage of the socket, removal of debridement and local medicament placement (Alvogyl‐Septodont) as an antiseptic and analgenic paste which contain Butamben, Jodoform, and Eugenol. Alvogyl easily adheres to the socket walls and gradually self‐eliminates by movement of patient tongue. Table 3 shows data related to dry socket 7 days after extraction in terms of reason for extraction.

**Table 3:** Reasons for the extraction of third molars of the lower jaw

	Dry socket	
	Yes (*n*=1)	No (*n*=29)
Pericoronitis	1 (100%)	17 (56.7%)
Caries	0	1 (3.3%)
Orthodontic indication	0	11 (36.7%)

## 4 DISCUSSION

Surgical extraction of the third molars of the lower jaw often results in postoperative symptomatology which influences daily obligations of patients for several days after surgery. The postoperative complications can hardly be predicted and significantly increase the probability for delayed rehabilitation. For that reason, surgical extraction of third molars is not a risk‐free intervention due to the possibility of occurring complications (Arita et al., 2005). For instance, Chaparro‐Avendano et al. (2005) reported pain as the most common complication within all complications after extraction of the third molars of the lower jaw. In the current study the aim was to compare the efficacy of ozone gas and CHX gel in terms of postoperative pain intensity and occurrence of dry socket.

Our results indicate that both, ozone gas on CHX gel, effectively prevent the serious postoperative pain, and the difference between them was not significant. The positive effect of ozone on the pain after surgical extraction of third molars was also shown by Kazancioglu et al. (2014) in their clinical trial on 60 patients, which resulted in significant reduction of pain and number of taken analgesic tablets in the postoperative period. Their study showed that ozone therapy was successful in the recovery period on the base of patient's questionnaire. Regarding the pain, ozone helps synthesize substances that interfere with pain reduction such as interleukins, leukotrienes, and prostaglandins. This justifies the results of our study of its effect on reducing/preventing severe postoperative pain. In case of pain occurrence, paracetamol analgesics or nonsteroidal anti‐inflammatory drugs (NSAIDS) are the most common drugs prescribed to patients to control postoperative pain, which basically in most cases is transient within 24–48 h after the intervention, but not including cases of dry socket development (Dionne, 1999; Seymour et al., 1985). In our work, ibuprofen 400 mg was selected for the management of postoperative pain, as in addition to the analgesic effect, it also has anti‐inflammatory properties with a positive effect on edema and trismus, which follow the surgical extraction of the third molar. In the group with ozone gas, it was noticed that the patients consumed less analgesics compared to the group of patients to whom CHX gel was applied.

Dry socket has been identified as one of the most challenging complications after surgical extraction of third molars. In our study in both groups, only one patient reported dry socket. The incidence rate is therefore within the limits reported in the literature (Hupp, 1998; Kolokythas et al., 2010). The frequency of dry socket has been the subject of many articles in the literature, but the lack of objective clinical diagnostic criteria is the reason for numerous discrepancies in these reports. The incidence of dry socket after normal tooth extractions is reported to be between 0.5% and 5% (Field et al., 1985). The incidence of dry socket after extraction of third molars varies from 1% to 37.5% and is about 10 times higher than in conventional extractions (Ritzau et al., 1992; Swanson, 1966).

Several discussions elevated the role of bacteria for the development of dry sockets. Such concept is based on reported results where the increase of dry socket was confirmed in patients with poor oral hygiene, in patients where infection was present as a pericoronitis, and in patients with advanced stage of periodontal disease (Rud, 1969). The impact of bacteria in the development of dry socket was explained by the reduction of the incidence of dry socket in trials that included the antibacterial preventive measures. Akota et al. (1998) demonstrated the reduction of the incidence of dry socket after the application of impregnated gauze with chlortetracycline, which showed a significant difference compared to the control group. Postoperative application of amoxicillin in combination with mouthwash also resulted in a significant reduction of dry socket after surgical extraction of third molars according to authors Delilbasi et al. (2002) Four other studies compared the efficacy of different types of antibiotics such as penicillin (Curran et al., 1974; Halpern & Dodson, 2007), clindamycin (Almendros‐Marqués et al., 2006), and metronidazole (Ritzau et al., 1992) in the prevention of dry socket after extraction.

Besides antibiotics, Haraji et al. (2013) investigated the effect of 0.2% CHX gel and obtained significant results in reducing the incidence of dry socket from 32.6% to 11.3% after the extraction of third mandibular molars. In addition to reducing the incidence of dry socket, their results also showed the positive effect of CHX in reducing postoperative pain. Lagares et al. (2006) encountered a reduction of dry socket from 30.76% in the control group to 17.64% in the experimental group, as well as positive impact on postoperative edema after one sole application of 0.2% CHX gel to the socket of impacted third molars. Similarly, was observed by Rubio‐Palau et al. (2015) after applying 0.2% CHX gel. The authors encountered a reduction in the incidence of dry socket by 22.2% compared to the placebo group. The results obtained on the efficacy of ozone gas in dry socket reduction are similar to the data of many authors who reported the positive effect in wound healing and stimulation of bone regeneration. Our study coincides with all of beforementioned studies, showing low incidence rates for dry socket and patients with low levels of pain.

Huth et al. (2009) investigated the efficacy of ozone in aqueous solution against periodontal pathogenic microorganisms along with CHX digluconate in solution form at different concentrations and concluded the efficacy in eliminating pathogenic microorganisms in the oral flora for both agents. In the clinical study on rabbits, Alpan et al. (2016) concluded that ozone gas stimulates bone tissue regeneration especially in the early stages of bone healing. Erdemci et al (2014). obtained positive histomorphometric results in terms of stimulation and formation of new alveolar bone after tooth extraction after application of systemic ozone to rabbits. Nevertheless, our findings are also significant for bringing innovation into clinical practice and the study design encompassed two groups that might prevent the development of postsurgical complications. The sample size that was used was relatively small, but within the limits of this study, we can conclude that the application of ozone gas and 1% CHX gel to the tooth alveola immediately after surgical extraction have similar capability for prevention of dry socket. Comparing the action of ozone and CHX on the parameters mentioned above, ozone gas is prioritized as an alternative premedication strategy in reducing the inflammatory symptoms and postoperative complications that accompany the surgical extraction of third molars. The advantage of this paper is that the same patient has been subjected to both test groups, one side belonged to Group 1 while the other side to Group 2. The patient was not aware of which side belonged to which group, in order not to reflect the therapy for subjective data.

As mentioned, our study had limitations. First, was very small sample size and as this was it was an observational prospective study, the patients were not randomized to two different groups, CHX and ozone group. Moreover, not a lot of additional factors (surgery‐related or patients‐related) that might affect dry socket prevention or occurrence were investigated. In our case only one patient developed the dry socket, which is according to the literature low percentage, so we are encouraged to conduct larger study to obtain more reliable results.

## 5 CONCLUSIONS

Ozone gas and CHX 1% gel are both efficient in decreasing postoperative symptoms and incidence rates of dry socket, but in comparison to each other, the use of ozone gas is showing better prevention capability. Based on the results obtained in the paper and research in the literature we can conclude that in the future, the focus should be designed on further clinical research, setting safe and well‐defined parameters and guidelines for routine use of ozone in prevention of postoperative complications such as occurrence of dry sockets.

## AUTHOR CONTRIBUTIONS

*Manuscript preparation and data collection*: Jehona Ahmedi and Zana Agani. *Data collection, data interpretation, and literature search*: Resmije Ademi Abdyli. *Study design and final approval of the manuscript*: Mergime Prekazi Loxha. *Data collection, data interpretation, and statistical analyses*: Vjosa Hamiti‐Krasniqi and Aida Rexhepi. *Statistical analyses and final approval of the manuscript*: David Stubljar.

## CONFLICT OF INTEREST

The authors declare no conflict of interest.

## Data Availability

Data are available upon request from the corresponding author.
